# Ferrostatin-1 improves acute sepsis-induced cardiomyopathy via inhibiting neutrophil infiltration through impaired chemokine axis

**DOI:** 10.3389/fcell.2024.1510232

**Published:** 2024-12-12

**Authors:** Jialin Li, Fang Xiao, Bingsen Lin, Zhilei Huang, Mingyue Wu, Huan Ma, Ruoxu Dou, Xiaodong Song, Zhongxing Wang, Changjie Cai, Xiangdong Guan, Jie Xu, Fu-Li Xiang

**Affiliations:** ^1^ Department of Critical Care Medicine, First Affiliated Hospital of Sun Yat-sen University, Guangzhou, China; ^2^ Institute of Precision Medicine, First Affiliated Hospital of Sun Yat-sen University, Guangzhou, China; ^3^ Department of Anesthesia, First Affiliated Hospital of Sun Yat-sen University, Guangzhou, China; ^4^ NHC Key Laboratory of Assisted Circulation and Vascular Diseases, Sun Yat-sen University, Guangzhou, China

**Keywords:** sepsis-induced cardiomyopathy, ferrostatin-1(Fer-1), neutrophil, chemokine, ferroptosis

## Abstract

**Introduction:**

Sepsis-induced cardiomyopathy is a common complication of sepsis and is associated with higher mortality. To date, effective diagnostic and management strategies are still lacking. Recent studies suggest that ferroptosis plays a critical role in sepsis-induced cardiomyopathy and ferroptosis inhibitor Ferrostatin-1 (Fer-1) improved cardiac dysfunction and survival in lipopolysaccharide (LPS) induced endotoxemia. However, the effects of Fer-1 in cardiac dysfunction in the early stages of cecal ligation and puncture (CLP) induced sepsis remains unclear. Our study aims to elucidate the role of Fer-1 in the acute phase of peritonitis sepsis induced cardiac injury.

**Methods and Results:**

CLP was used to induce peritonitis sepsis in mice. Pretreatment of ferroptosis inhibitor ferrostatin-1 (Fer-1) was used in the in vivo models. Survival was monitored for 48h. Cardiac function and histology were analyzed 6h after surgery. We found that ejection fraction (EF) remained normal at 6h after CLP, but the contractility detected by cardiac muscle strain analysis was significantly reduced, along with increased immune cell infiltration. Pretreating the CLP mice with 5 mg/kg Fer-1 significantly reduced mortality. At 6h after CLP, ferroptosis key regulator Gpx4, cardiac iron and malonaldehyde (MDA) did not change, but ferroptosis marker gene expression increased. Fer-1 treatment showed beneficial effects in cardiac function, less myocardial inflammatory cytokine expression and significantly inhibited immune cells, especially neutrophil infiltration in the heart. Consistently, expression of neutrophil associated chemokines (Ccrl2, Cxcl2, Cxcl3 and Cxcl5) as well as extracellular matrix (ECM) degradation enzymes (Adamts1, Adamts4, Adamts9 and Mmp8) significantly decreased in Fer-1 pre-treated CLP heart.

**Conclusion and Discussion:**

Our findings suggest that Fer-1 inhibits neutrophil infiltration in early sepsis by disrupting the chemokine axis, highlighting its potential as a therapeutic option to manage acute immune overactivation in early stages of sepsis-induced cardiomyopathy.

## Introduction

Sepsis is a dysregulated host response to infection that can lead to life-threatening organ failure. Cardiac dysfunction is commonly observed in patients with severe sepsis and septic shock, with an incidence reported to be between 10% and 70% (Shvilkina and Shapiro, 2023). Sepsis-induced cardiomyopathy was initially defined by a reversible depression in EF with ventricular dilation (Parker et al., 1984). It significantly exacerbates the risk of multiorgan failure and is associated with at least a two-fold increase in mortality (Parrillo et al., 1990; Martin et al., 2019). Despite advancements in intensive care unit medicine, no clear diagnoses standard and effective interventions have been established. Understanding the progression and mechanisms of sepsis-induced cardiomyopathy is crucial for developing effective diagnostic and therapeutic strategies.

Inflammation and immune dysfunction are the main mechanisms underlying the occurrence and development of sepsis (Leligdowicz et al., 2014; Giamarellos-Bourboulis et al., 2024; Zhang and Ning, 2021; van der Poll et al., 2021). Sepsis is initiated with an uncontrolled infection and progresses with a hyperactivated immune response to pathogen infection at early stage, which is characterized by overwhelmed immune cell activation and cytokine storm release (Feuerecker et al., 2018; Reyes et al., 2020; Rittirsch et al., 2008). The impaired cardiac function and sustained cardiac injury at early stage in sepsis are associated with direct cardio-depression effects from inflammatory cytokines and damage from infiltrated immune cells (Vincent et al., 1992; Schulte et al., 2013). Activated circulating neutrophils are recruited to myocardial tissue through the action of chemokines and release proinflammatory cytokines (Huang et al., 2022; Cao et al., 2024). Thus, controlling pro-inflammatory cytokine expression and preventing excessive infiltration of inflammatory immune cells are potential strategies for sepsis induced cardiomyopathy management.

As one of the most metabolically active organs, the heart is highly susceptible to inflammatory insults during the early phases of sepsis. The systemic inflammatory response triggers a cascade of cytokine production, which in turn leads to oxidative stress, a mechanism strongly implicated in the pathogenesis of sepsis-induced cardiomyopathy. Ferroptosis, a form of programmed cell death intricately linked to oxidative stress and iron metabolism (Cui et al., 2024) (Wang et al., 2024), has emerged as a potential contributor to many forms of cardiac injury, including ischemic cardiac injury (Gao et al., 2015; Li et al., 2019), cardiac hypertrophy (Bi et al., 2024; Shi et al., 2022), diabetic cardiomyopathy (Zhao et al., 2024; Wang et al., 2022) and doxorubicin-induced cardiotoxicity (Fang et al., 2019), and sepsis induced cardiomyopathy (Li et al., 2020; Xiao et al., 2021; Wang et al., 2020). Inhibiting ferroptosis by Fer1 can alleviate cardiac dysfunction in LPS-induced endotoxemia (Fang et al., 2019). However, the effects of Fer-1 in early cardiac dysfunction and systemic inflammation induced by sepsis model like CLP have not yet been reported.

In our study, we aim to elucidate the early changes in cardiac function by echocardiography strain analysis in CLP, a murine model of sepsis, and investigate the effects of Fer-1 in sepsis related acute cardiac injury. Additionally, we seek to better understand the progression and mechanisms of early-stage cardiac injury during sepsis and discover potential early intervention to improve acute sepsis induced cardiac dysfunction.

## Results

### Cardiac dysfunction was observed at the early stage of CLP-induced sepsis

**FIGURE 1:**
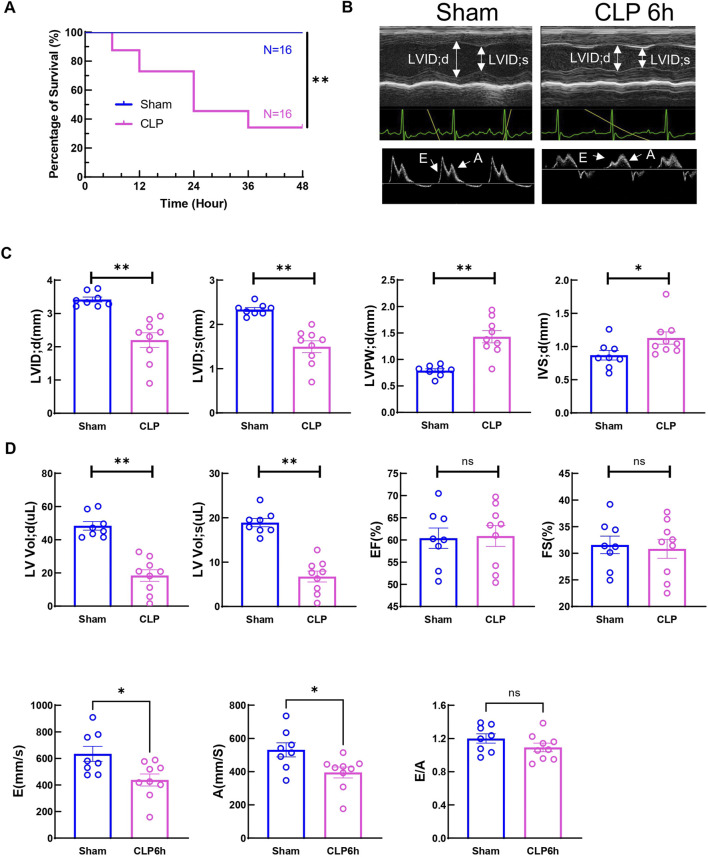
Two-day mortality and Cardiac function at 6h after CLP induced sepsis. **(A)** Survival was monitored from 0 to 2 days post-CLP. Statistical significance was determined using Log-rank (Mantel-Cox) test: **p < 0.01 vs Sham. **(B)** Cardiac function was measured 6 h post-surgery via echocardiography. Representative M-mode tracing images and Doppler echocardiography tracing images are shown. Upper panels, arrows indicate the left ventricular internal diameter in diastole (LVIDd) and systole (LVIDs). Lower panels, arrows indicate E wave and A wave. **(C)** Cardiac Echocardiographic indicators (LVIDd, LVIDs, LVPWd, IVSd, LV vold, LV vols), cardiac systolic function (EF, FS) was measured 6 h post-surgery via echocardiography. **(D)** Cardiac diastolic function (E wave, A wave and E to A ratio) was measured 6 h post-surgery via echocardiography. Data are presented as mean ± SEM. Statistical significance was determined using Mann-Whitney U tests: *p < 0.05 vs sham. **p < 0.01 vs sham. N = eight to nine mice/group. LVIDd: left ventricular end-diastolic diameter; LVIDs: left ventricular end-systolic diameter; LVPWd: left ventricle posterior wall thickness in diastole/systole; IVSd: interventricular septum thickness in diastole; LV Vol **(D)** left ventricular end diastolic volume; LV Vol s: left ventricular end-systolic volume; EF: ejection fraction; FS: fractional shortening.

**FIGURE 2:**
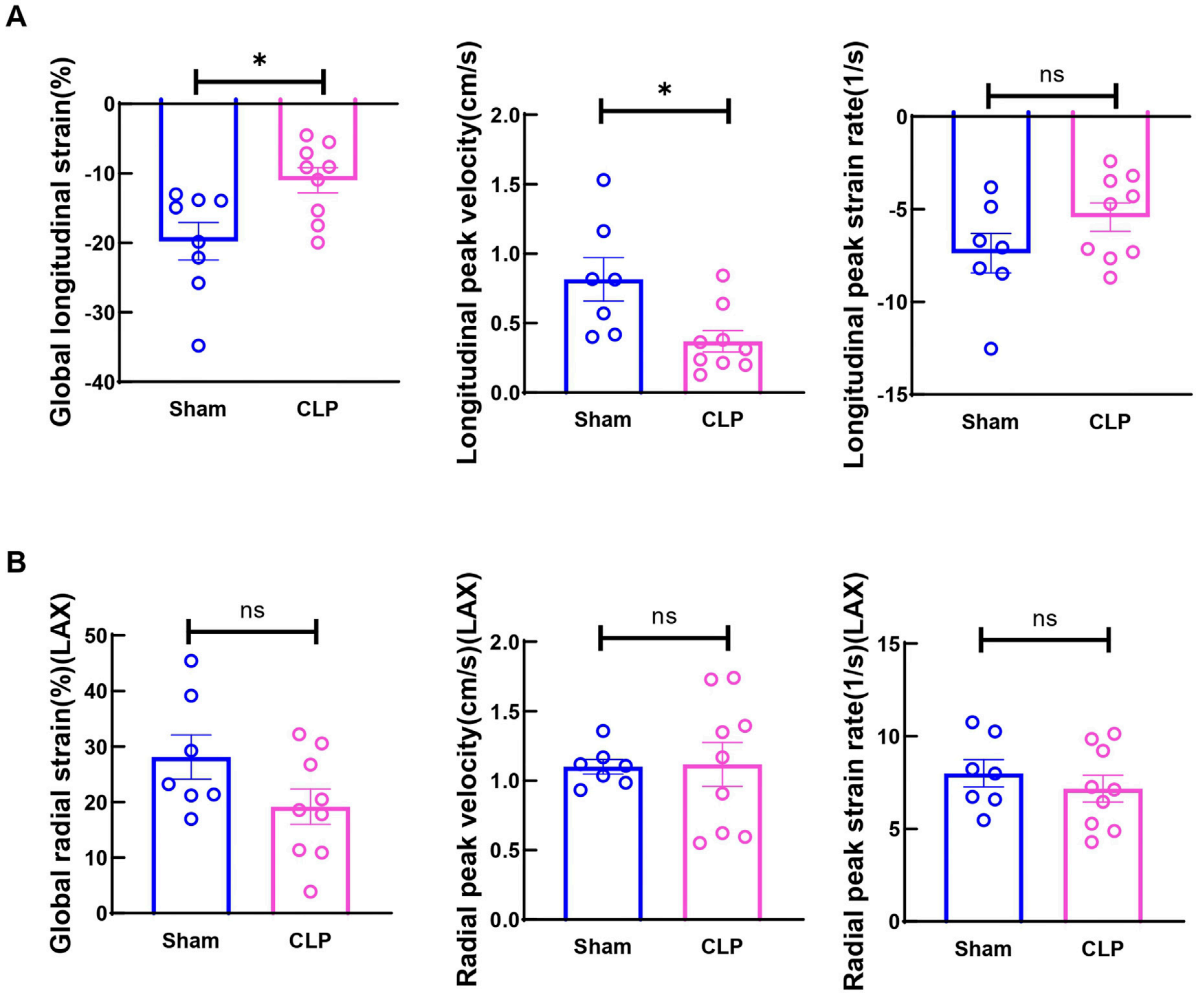
Cardiac function measured by strain analysis at 6h after CLP induced sepsis. **(A)** Global longitudinal strain (GLS) was analyzed in the long-axis imaging plane. Longitudinal peak systolic strain rate, and longitudinal peak systolic velocity were acquired in the long axis imaging plane. **(B)** Global radial strain was analyzed in the long axis (LAX) imaging plane. Radial systolic peak velocity and radial systolic peak strain rate were acquired in the long axis imaging plane. Data are presented as mean ± SEM. Statistical significance was determined using Mann-Whitney U tests: *p < 0.05 vs sham. N = seven to nine mice/group.

CLP, a model of acute polymicrobial septic peritonitis, was employed to study acute cardiac dysfunction in sepsis. Mice with CLP in our study showed a mortality rate of about 70% after 48 h (n = 16, Figure 1A) and most of the death occurred after 6h, therefore this time point was chosen to study the early changes in cardiac function and injury. The traditional two-dimensional cardio echography showed significantly decreased LVID; d, LVID; s, and increased LVPW; d and IVS; d (Figures 1B, C), indicating smaller LV chamber volume and thicker LV wall at 6 h after CLP. However, EF and fraction shortening (FS) showed no change at this time point. The mitral E and A wave measured at 4-chamber view both decreased significantly, but E/A ratio did not change (Figures 1B, D). Considering that EF and mitral flow is load-dependent parameters and have been reported to be unreliable due to the highly dynamic nature of circulating volume in sepsis conditions (Parker et al., 1984; Jardin et al., 1999), cardiac muscle strain analysis was used to measure loading independent parameters (see Supplementary Videos S1–3). Consistent with clinical findings (De Geer et al., 2015; Lanspa et al., 2015), myocardial global longitudinal strain (GLS) and longitudinal peak velocity (LPV) were significantly impaired in mice 6 h after CLP (Figure 2A), while radical parameters showed no differences (Figure 2B). These results demonstrated that cardiac muscle strain analysis such as GLS can detect early cardiac dysfunction in sepsis model.

### Ferroptosis marker Gpx4, cardiac iron and MDA showed no change in heart tissue at 6 h after CLP

**FIGURE 3:**
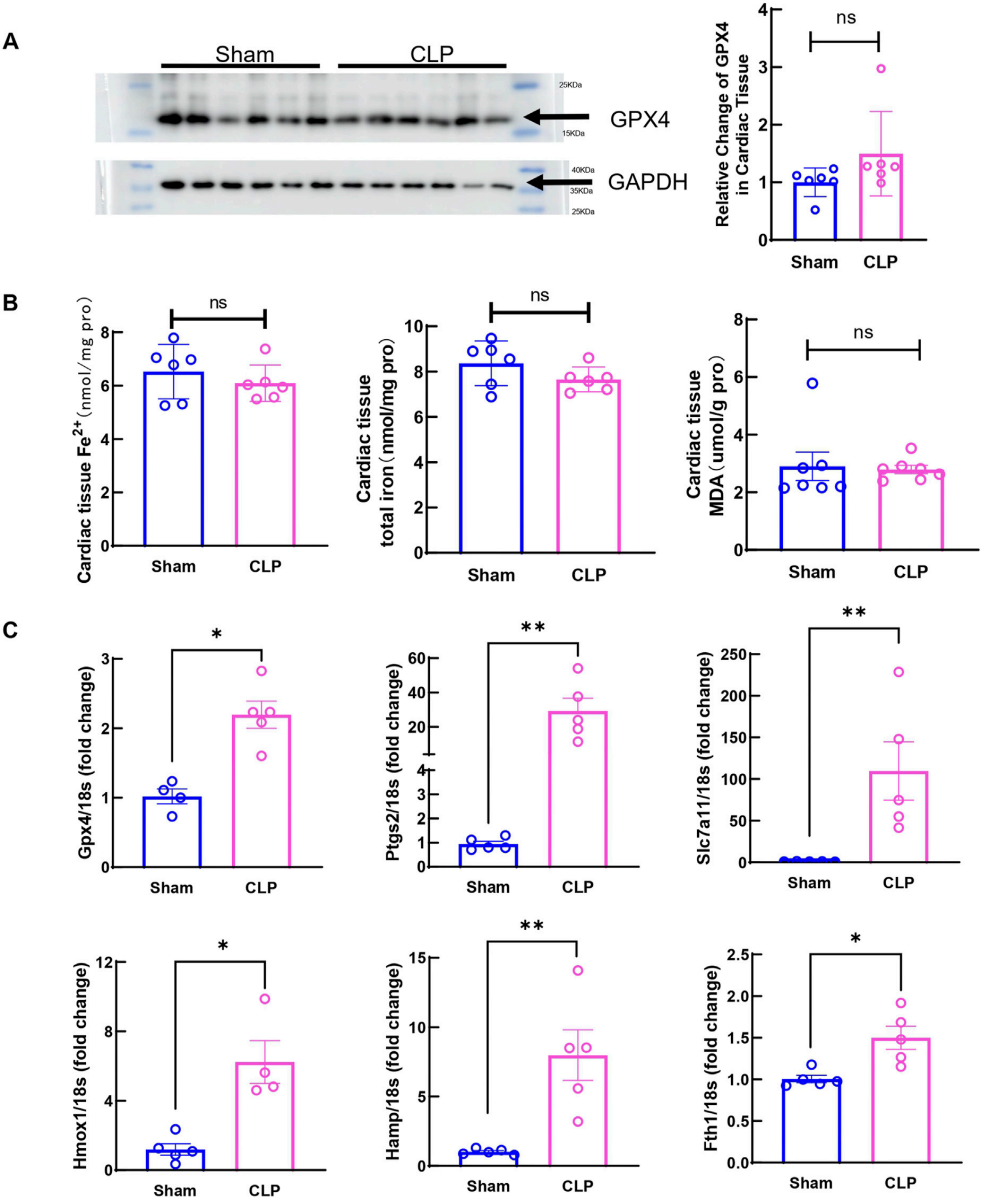
Ferroptosis markers in cardiac tissue at 6h after CLP induced sepsis. **(A)** Cardiac Gpx4 expression was determined by Western blot analysis. N = 6/group. **(B)** Serum iron, cardiac nonheme iron content and cardiac MDA levels were measured in the Sham group and CLP group. N = 6–8/group. **(C)** mRNA was isolated from LV tissue. Expression of ferroptosis markers in CLP group was detected by qRT-PCR in relative to Sham group. N = 4–5/group. Data are presented as mean ± SEM. Statistical significance was determined using Mann-Whitney U tests: *p < 0.05 vs sham. **p < 0.01 vs sham.

Studies have shown that significantly increased ferroptosis associated changes are observed in myocardium at 12h and 24 h after sepsis onset in animal models (Li et al., 2020; Wang et al., 2020). Moreover, transcriptome analysis also revealed that ferroptosis related signaling are upregulated in hearts from patients who died from systemic sepsis (Song et al., 2023). However, whether ferroptosis is involved in early cardiac injury in sepsis is still unknown. Moreover, the mechanism of ferroptosis-mediated, sepsis-induced cardiac dysfunction is not fully understood. To find out if ferroptosis contributes to early cardiac dysfunction and injury, ferroptosis markers were measured. The protein level of the key ferroptosis regulator Gpx4 showed no change at 6 h after CLP (Figure 3A), Moreover, no changes in myocardial iron and MDA were observed (Figure 3B), suggesting that at this stage, ferroptosis did not increase in local cardiac tissue. Apoptosis was also evaluated by TUNEL staining in CLP hearts. No obvious TUNEL signals were detected at 6 h post-CLP (Supplementary Figure S1). But expressions of Gpx4, Ptgs2, Slc7a11, Hmox1, Hamp and *F*th1 mRNA significantly changed in the heart (Figure 3C). These data showed that ferroptosis related signaling is not yet activated in myocardium at 6 h after CLP induced sepsis, suggesting that ferroptotic cell death might not be directly responsible for early cardiac dysfunction and injury in our CLP model.

### Ferroptosis inhibitor Fer-1 improved 2-day survival after CLP and alleviated early cardiac dysfunction at 6 h after CLP

**FIGURE 4:**
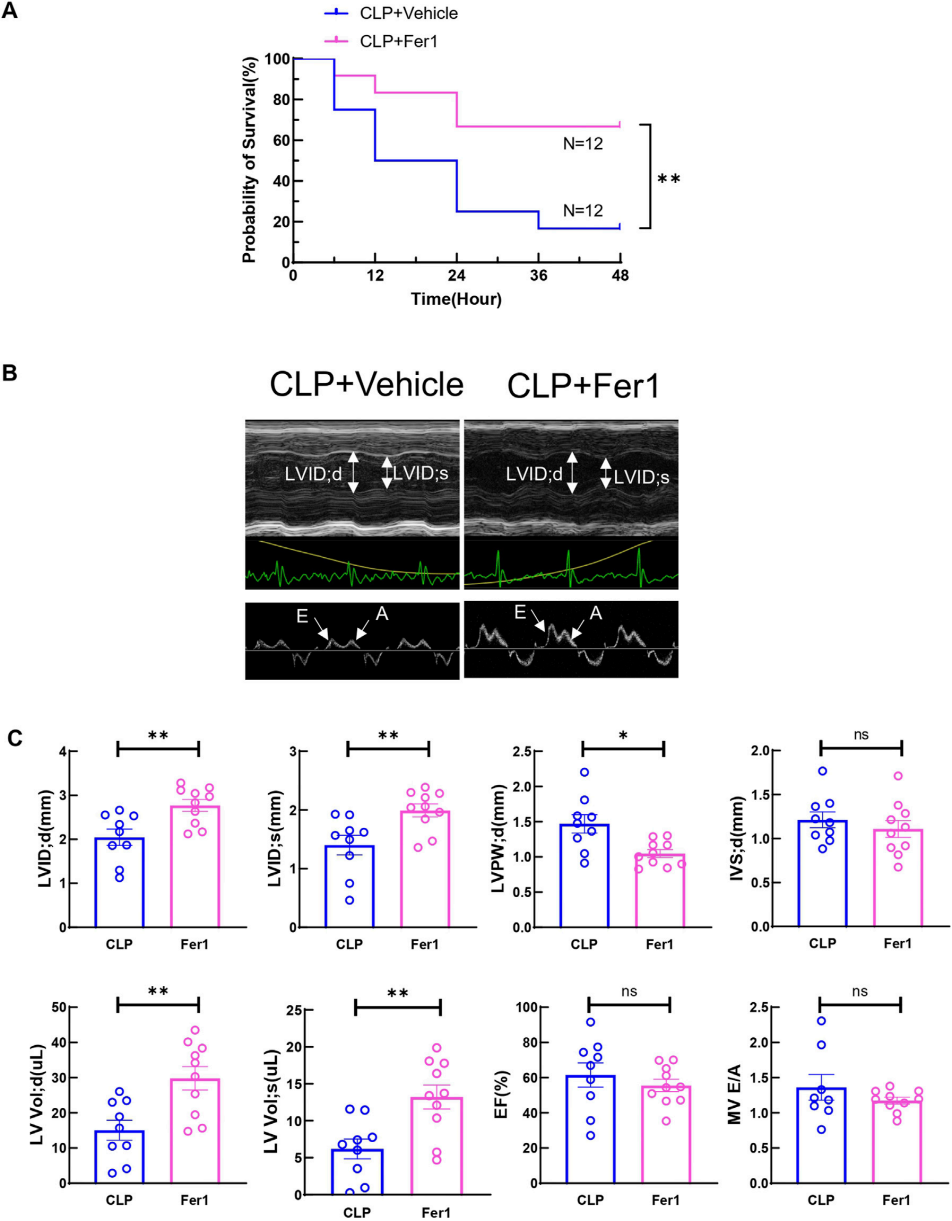
Fer-1 rescued CLP induced sepsis 2-day mortality and partially improved cardiac function at 6h after CLP induced sepsis. **(A)** Survival was monitored from 0 to 2 days post-CLP surgery in CLP + Vehicle (CLP) group and CLP + Fer-1 (Fer-1) group. Statistical significance was determined using Log-rank (Mantel-Cox) test: **p < 0.01 vs CLP. **(B)** Cardiac function was measured 6 h post-surgery via echocardiography. Representative M-mode tracing images and Doppler echocardiography tracing images are shown. Upper panels, arrows indicate the left ventricular internal diameter in diastole (LVIDd) and systole (LVIDs). Lower panels, arrows indicate E wave and A wave. **(C)** Cardiac Echocardiographic indicators (LVIDd, LVIDs, LVPWd, IVSd, LV vold, LV vols), cardiac systolic function (EF) and diastolic function (E to A ratio) was measured 6 h post-surgery via echocardiography. Data are presented as mean ± SEM. Statistical significance was determined using Mann-Whitney U tests: *p < 0.05 vs CLP. **p < 0.01 vs CLP. N = 9–10 mice/group.

**FIGURE 5:**
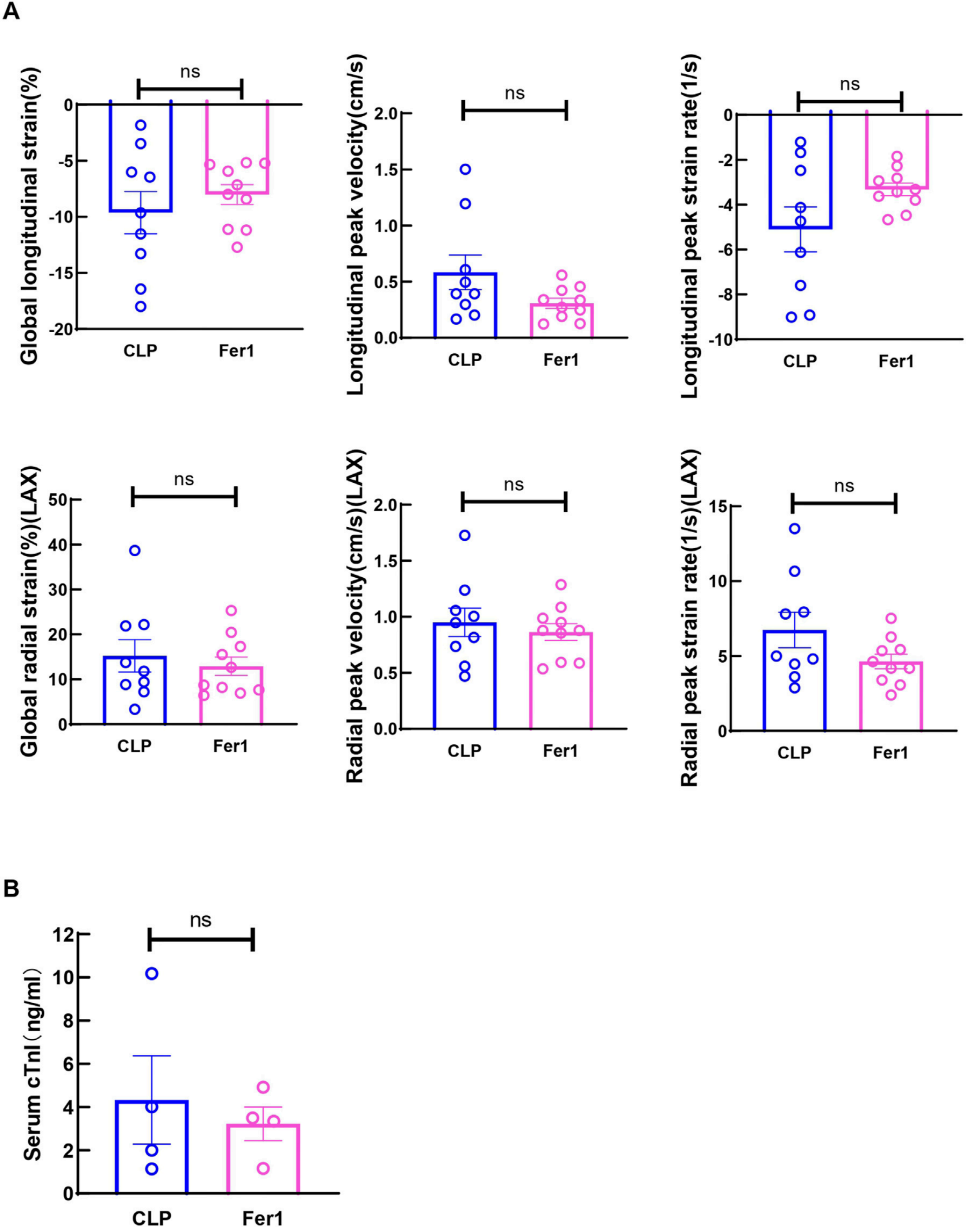
Fer-1 has no impact on strain related cardiac performance at 6h after CLP induced sepsis. **(A)** Global longitudinal strain (GLS), analyzed in the long-axis imaging plane. Longitudinal peak systolic strain rate, and longitudinal peak systolic velocity, acquired in the long axis imaging plane. Global radial strain, analyzed in the long axis (LAX) imaging plane. Radial systolic peak velocity and radial systolic peak strain rate, acquired in the long axis imaging plane. **(B)** Serum cardiac muscle injury biomarker cTNI was measured 6 h after CLP. Data are presented as mean ± SEM. Statistical significance was determined using Mann-Whitney U tests. N = 9–10 mice/group.

Fer-1 pre-treatment has been shown to improve cardiac function at 12 h after LPS injection and 1-week survival in LPS induced endotoxemia in mice (Li et al., 2020). In our CLP induced sepsis model, pre-treating the mice with 5 mg/kg Fer-1 significantly improved 2-day survival compared to the vehicle group (n = 12, Figure 4A). At 6 h after CLP, Fer-1 pre-treatment group showed significantly increased LVID; d, LVID; s, and decreased LVPW; d (Figures 4B, C). Cardiac muscle strain analysis was also performed to evaluate load-independent parameters of cardiac function, specifically GLS and LPV. Both CLP and Fer-1 group showed impaired muscle strain, but no significant differences were observed in GLS and LPV between CLP and Fer-1-treated group (Figure 5A). Accordingly, no difference was observed in serum cardiac muscle injury biomarker cTNI between CLP and Fer-1-treated group (Figure 5B). Collectively, these results further confirmed that Fer-1 could partially improve cardiac function in early sepsis, which might contribute to the significantly improved 2-day survival.

### Fer-1 significantly decreased pro-inflammatory cytokine expression and affected some ferroptosis marker gene expression in myocardium at 6 h after CLP

**FIGURE 6:**
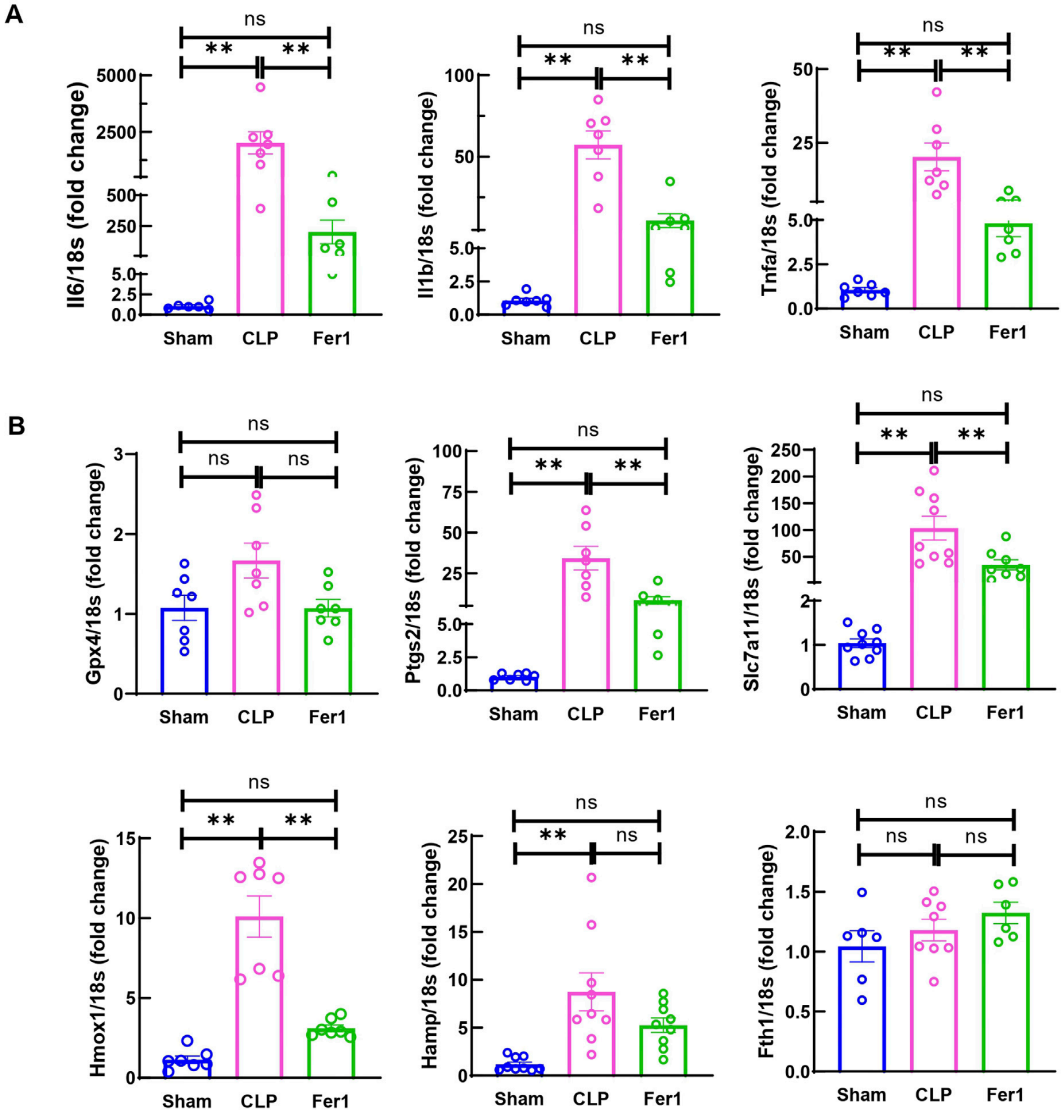
Fer-1 decreased inflammatory cytokine and ferroptosis marker expression in hearts of CLP induced sepsis. mRNA was isolated from LV tissue at 6 h after CLP. **(A)** Expression of inflammatory cytokine in CLP and Fer-1 was detected by qRT-PCR in relative to Sham group. **(B)** Expression of ferroptosis markers in CLP and Fer-1 was detected by qRT-PCR in relative to Sham group. N = 6–9/group. Data are presented as mean ± SEM. Statistical significance was determined using one-way ANOVA followed by Bonferroni’s multiple comparisons tests: *p < 0.05. **p < 0.01.

To investigate Fer-1’s effects in acute sepsis induced cardiomyopathy, pro-inflammatory cytokines and key ferroptosis marker genes were measured. Interestingly, at 6 h after CLP, Fer-1 pre-treated group displayed significantly reduced myocardial IL-6, IL-1β and TNFα gene expression (Figure 6A). Moreover, three (Ptgs2, Slc7a11 and Hmox1) out of the six CLP affected ferroptosis markers were significantly inhibited by Fer-1 pre-treatment in CLP hearts (Figure 6B). These results confirmed that Fer-1 pre-treatment decreases inflammation during early sepsis and regulates myocardial ferroptosis signaling.

### RNAseq analysis revealed that Fer-1 mainly affected chemokine axis, ECM degradation and endothelial regulators in myocardium at 6 h after CLP

**FIGURE 7:**
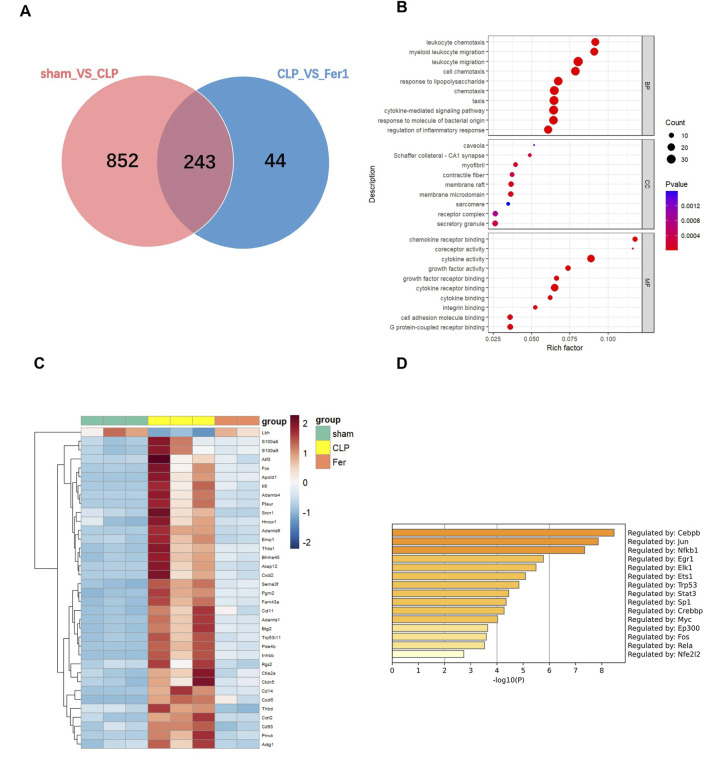
Transcriptome analysis of heart tissues from sham, CLP and CLP + Fer-1 mice 6h after surgery. mRNA was isolated from LV tissue. **(A)** Venn diagram showing the number of shared genes in the comparison: pink, sham group vs. CLP group, blue, CLP group vs. Fer-1 group. **(B)** GO Term analysis of shared differentially regulated genes. **(C)** Heat map of normalized count values for selected genes. **(D)** GO-TRRUST enrichment of transcriptional regulators. Analyses were performed using Metascape.com (version 3.5.20240901).

**FIGURE 8:**
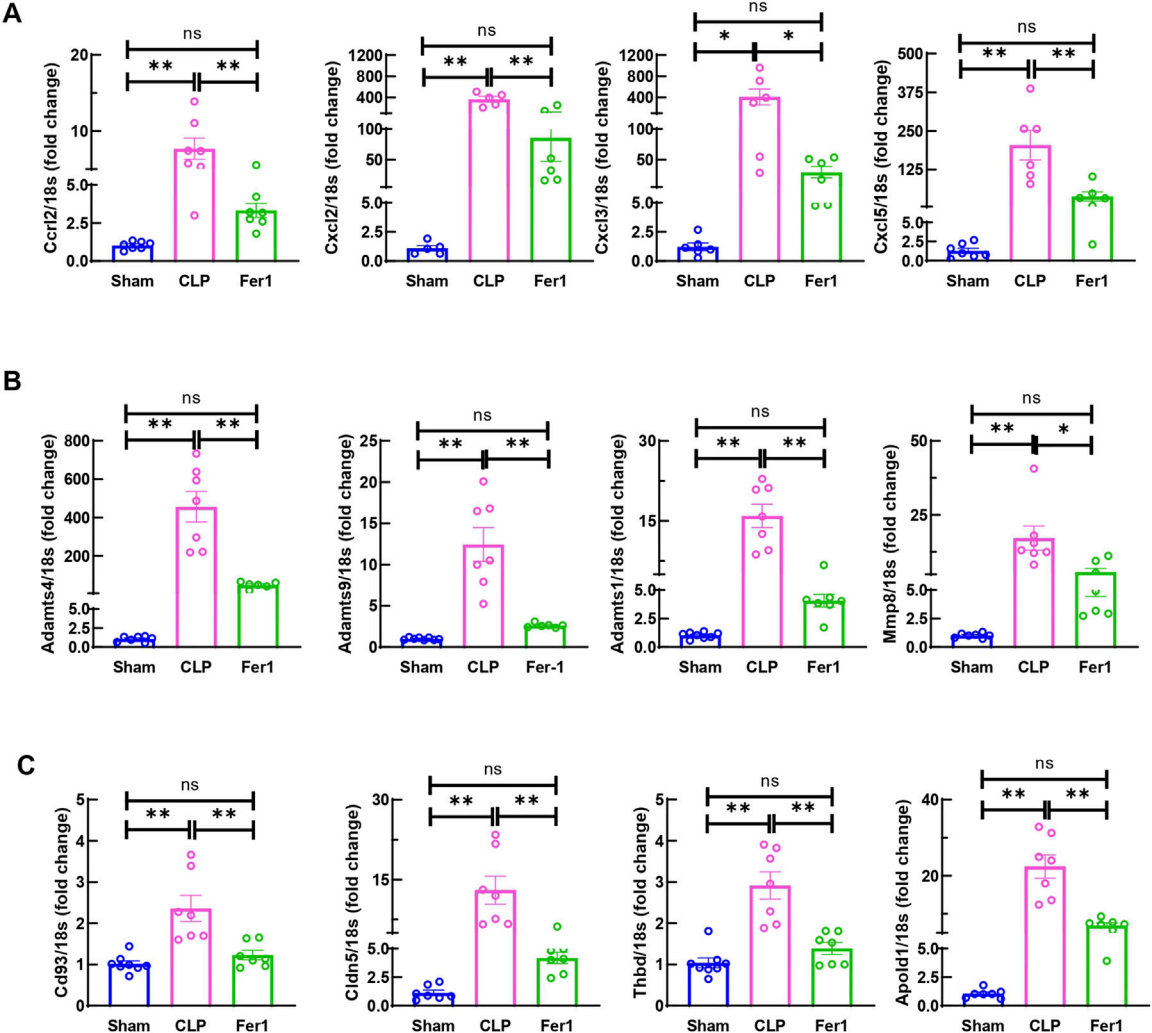
Fer-1 decreased chemokine, ECM degradation and endothelial dysfunction related gene expression in hearts of CLP induced sepsis. mRNA was isolated from LV tissue at 6 h after surgery. **(A)** Expression of chemokine in CLP and Fer-1 was detected by qRT-PCR in relative to Sham group. **(B)** Expression of ECM degradation related gene in CLP and Fer-1 was detected by qRT-PCR in relative to Sham group. **(C)** Expression of endothelial dysfunction related gene in CLP and Fer-1 was detected by qRT-PCR in relative to Sham group. N = 4–8/group. Data are presented as mean ± SEM. Statistical significance was determined using one-way ANOVA followed by Bonferroni’s multiple comparisons tests: *p < 0.05. **p < 0.01.

To further clarify Fer-1’s effects in acute sepsis induced cardiomyopathy, transcriptome of sham, CLP and Fer-1 treated CLP hearts were analyzed at 6 h after CLP (N = 2–3). At FDR-corrected p-value of 0.05 and fold change (FC) of 2, 773 genes were detected as upregulated in CLP-induced mouse hearts, whereas 332 genes were downregulated (Supplementary Figure S2A). Functional enrichment analysis revealed that the upregulated genes in CLP-induced acute septic hearts were primarily involved in activation of innate immune response, chemokine activity and cytokine-mediated signaling pathway (Supplementary Figure S2B). Using the same selection criteria, only 50 genes were detected as upregulated whereas 237 genes were downregulated in CLP + Fer-1 mouse hearts (Supplementary Figure S2C). After cross-comparing the differentially expressed genes, 243 genes are commonly regulated in the heart by CLP and Fer-1 (Figure 7A), mostly involved in chemokine receptor binding, leukocyte chemotaxis and cytokine activity as revealed by GO analysis (Figure 7B). After strengthening the selection criteria using p-value of 0.05 and absolute FC of two plus FPKM ≥ 30 (high gene expression abundancy) in CLP or Sham group, 36 top regulated genes were selected (Figure 7C). The transcription factors associated with these function changes revealed by GO-TRRUST enrichment of transcriptional regulators include Cebpb, Jun, and NFκb (Figure 7D), which are master regulators for inflammatory cytokines and chemokines. Interestingly, after gene validation, Fer-1 treatment was found to significantly inhibit expression of chemokine axis (Cxcl2, Cxcl3, Cxcl5 and Ccrl2) (Figure 8A), ECM degradation regulators (Adamts1, Adamts4, Adamts9 and Mmp8) (Figure 8B) and endothelial cell migration regulators in CLP hearts (Cd93, Cldn5, Thbd and Apold1) (Figure 8C). These results suggested that Fer-1 pre-treatment might inhibit immune cell infiltration via suppression of chemokine signaling, ECM degradation and endothelial dysfunction in early stage of sepsis.

### Fer-1 inhibited neutrophil infiltration in myocardium at 6 h after CLP

**FIGURE 9:**
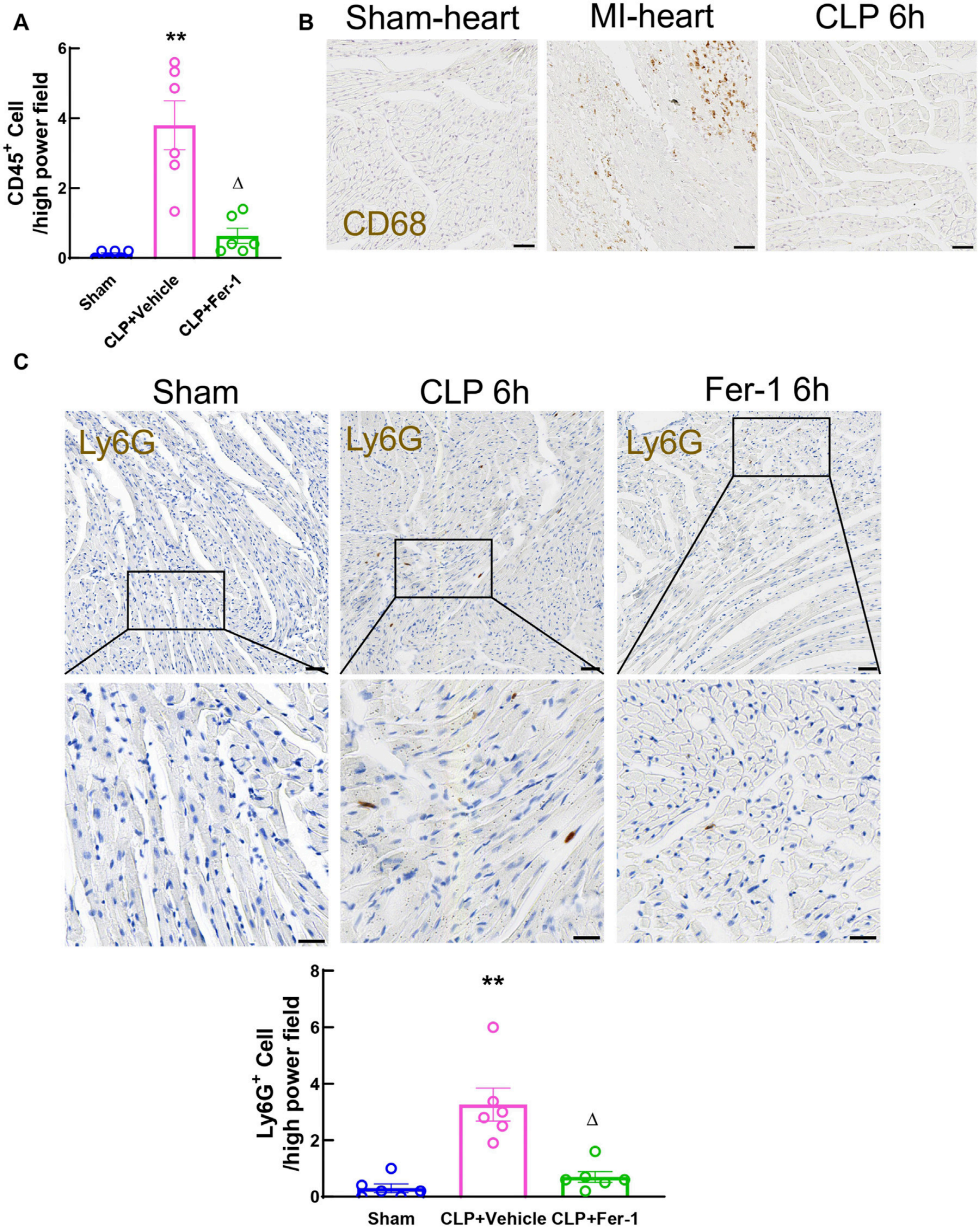
Myocardial immune cell infiltration at 6h after CLP induced sepsis. **(A)** Quantitative analyses of Cd45-stained heart sections from Sham, CLP and Fer-1 group. **(B)** Representative images of Cd68-stained heart sections from sham, MI and CLP mice. **(C)** Representative images and quantitative analyses of Ly6G-stained heart sections from Sham, CLP and Fer-1 group. Data are presented as mean ± SEM. Statistical significance was determined using one-way ANOVA followed by Bonferroni’s multiple comparisons tests: *p < 0.05 vs sham. **p < 0.01vs sham. ^*Δ*^P < 0.05 vs CLP. N = 6/group.

Inflammation is the trigger of the occurrence and development of sepsis. In CLP model, the systemic inflammation is initiated from acute polymicrobial septic peritonitis (Blanco et al., 2008; Brun-Buisson et al., 2004). Activated immune cells circulate in the blood stream and infiltrate myocardium and cause further organ injury (Du et al., 2022; Nong et al., 2023). To evaluate the immune cell infiltration, myocardial CD45^+^ cells were stained and quantified in heart tissue sections at 6 h after CLP. The density of CD45^+^ cells in CLP heart tissue at 6 h significantly increased compared to sham, however Fer-1 pre-treatment abolished this increase (Figure 9A). To clarify the type of immune cells infiltrated into the myocardium at this time point, Ly6G and CD68 were used to mark neutrophil and macrophage populations in the tissue sections respectively. CD68 staining showed very few signals in CLP heart sections while strongly stained the macrophage in the infarcted heart after myocardial infarction (positive control for CD68 staining) (Figure 9B). On the other hand, Ly6G staining showed a significantly increased neutrophil population in the CLP myocardium compared to sham, but this was significantly decreased in Fer-1 group (Figure 9C). These data showed that Fer-1 pre-treatment has beneficial effects in acute sepsis induced cardiomyopathy via inhibiting immune cell infiltration to the heart, especially neutrophils.

## Discussion

Sepsis induced cardiomyopathy is caused by systemic inflammatory response in sepsis and is associated with increased mortality in clinics (Charpentier et al., 2004; Beesley et al., 2018). However, despite significant progress in medicine, a standardized diagnosis and treatment is still lacking for sepsis induced cardiomyopathy especially at early stage of sepsis (Huang et al., 2013; Ehrman et al., 2018). Recently, myocardial ferroptosis has been shown to play an important role in sepsis induced cardiomyopathy and ferroptosis inhibitor Fer-1 pre-treatment improves survival in sepsis animal models (Li et al., 2020; Wang et al., 2023), but the underlying mechanisms are still not fully understood. In the current study, mouse CLP model was employed to investigate the early changes in cardiac function and the therapeutic effects of Fer-1 for acute sepsis induced cardiomyopathy. The major findings are: 1) At 6 h after CLP, EF remained normal but cardiac muscle strain index declined. 2) Ferroptosis regulator Gpx4, cardiac iron and MDA did not change at this early time point but ferroptosis marker genes increased. 2) Fer-1 pre-treatment significantly improved 2-day survival, partially alleviated cardiac dysfunction at 6 h after CLP. 3) Fer-1 pre-treatment significantly inhibited immune cells, especially neutrophil infiltration in the myocardium at 6 h after CLP through suppression of chemokines and extracellular matrix (ECM) degradation enzymes. These data revealed a therapeutic potential of Fer-1 for acute sepsis-induced cardiomyopathy via regulation of myocardial neutrophil infiltration.

Sepsis-induced cardiomyopathy is commonly studied using CLP or LPS animal model. CLP model in mice can induce acute polymicrobial septic peritonitis and is commonly used to mimic sepsis condition in clinic, including sepsis induced cardiac dysfunction. Sepsis-induced cardiac dysfunction was initially defined by reversable abnormal systolic function, specifically, a reduced LVEF (Parker et al., 1984; Boissier et al., 2017). However, in the past decade, clinic observations have shown that LVEF is highly variable and loading dependent, and not always associated with worse outcome in sepsis patients (Vieillard-Baron et al., 2008; Sanfilippo et al., 2018). Newly developed echocardiography technology using speckle tracing to visualize the cardiac muscle strain, which has been shown to be loading independent and better indicating the severity of the cardiomyopathy in sepsis (Orde et al., 2014; Basu et al., 2012). In current study, traditional two-dimensional echocardiography and cardiac muscle strain analysis were both used to evaluate the cardiac function in an acute CLP mouse model. Consistent with the findings in clinic, LVEF were maintained in normal range at 6 h after CLP, but the muscle stain analysis showed significantly decreased cardiac function, as demonstrated by less negative number of GLS. Sepsis-induced cardiac muscle dysfunction is a complex interplay of many pathological changes including inflammation, metabolic dysfunction, and oxidative stress. In the early phase of sepsis, myocardial injury can be confirmed by impaired cardiac muscle function as indicated by the strain analysis, which is consistent with the decreased LVID and LV volume. However, in clinics, the EF and cardiac output in sepsis patient are more likely maintained in normal range (Shvilkina and Shapiro, 2023; Boissier et al., 2017; Vieillard-Baron et al., 2008), which is consistent with what we have observed in our study. The underlying mechanisms might include sympathetic and parasympathetic system compensation by increased preload or altered vascular resistance which could maintain EF and FS within normal ranges to preserve cardiac output. These compensation mechanisms will eventually fail as the disease progresses, leading to decreased EF as shown by several studies using CLP or LPS model at 12 h or 24 h after the disease onset (Li et al., 2020; Wang et al., 2020).

Recently, ferroptosis has been shown to be involved in sepsis-induced cardiomyopathy. Significantly changed ferroptosis markers involving Gpx4 (Liu et al., 2024), Ptgs2, malonaldehyde (MDA), and cardiac iron have been found in LPS (Li et al., 2020) or CLP (Xiao et al., 2021; Wang et al., 2020) model 12 h pr 24 h after disease onset. Interestingly, our data show that in early time point (6 h) after CLP induced-sepsis, no changes were found in myocardial Gpx4 protein level, cardiac iron and MDA, suggesting that the myocardial ferroptosis signaling might not be the major drivers for the pathological changes in the early phase of sepsis-induced cardiomyopathy in mice. However, the observed mRNA upregulation of ferroptosis-related factors, including Ptgs2, Hmox1 and other genes at this early time point, could represent an initial cellular response to oxidative stress, inflammation, or disrupted iron homeostasis triggered by sepsis (Hoffman et al., 2019). This transcriptional activation may serve as a trigger for ferroptosis processes that manifest more distinctly over time and contribute to myocardial injury at later stages of sepsis (Li et al., 2021).

Ferroptosis inhibitor Fer-1 pre-treatment has been reported to improve survival and cardiac function in LPS induced endotoxemia model (Li et al., 2020; Xiao et al., 2021). Fer-1 was originally screened from small molecule libraries based on erastin-induced ferroptotic cell death assay (Dixon et al., 2012). Anti-ferroptotic activity of Fer-1 is due to its scavenging of alkoxyl radicals by ferrous iron from lipid hydroperoxides (Miotto et al., 2020). In addition, Fer-1 also exerts an anti-inflammation effect in acute ischemia reperfusion injury in kidney (Deng et al., 2024), liver (Yamada et al., 2020) (Lin et al., 2024) and heart (Li et al., 2019) via decreased IL-6, IL-1β and TNFα expression, as well as immune cell infiltration. In the LPS mouse model, Fer-1 pre-treatment decreases TNFα and IL-1β mRNA expression as well as macrophage infiltration in the myocardium 12 h after LPS injection (Li et al., 2020). However, the effects of Fer-1 in acute CLP sepsis induced cardiomyopathy are not clear. In our study, we found that Fer-1 pre-treatment improved CLP induced left ventricle chamber volume changes at early time point, but not cardiac muscle contractility as detected by strain analysis. However, Fer-1 pre-treatment significantly decreased 2-day mortality after CLP, which is consistent with previous reports (Li et al., 2020; Xiao et al., 2021). Regarding the disconnect between ventricle structure and cardiac muscle functional performance at early sepsis after Fer-1 treatment, we believe this observation suggests the beneficial effects of Fer-1 at early stage of sepsis might be due to the systemic inflammation regulation but not the direct impact on ferroptosis associated pathways in cardiac muscle injury. We have evidence that the level of ferroptosis associated changes have not significantly elevated at this early time point yet, which may explain why Fer-1 showed no effects on rescuing cardiac muscle injury at this early stage. Muscle contractile function as indicated by GLS and LPV might rely more on myocardial pathological stimulation from direct impairment of PAMP and other factors released from peritonitis into the circulation at early sepsis. It is also possible that the timeframe of our study was insufficient to observe functional improvements by Fer-1 treatment. A few studies have shown that Fer-1 treatment is able to improve EF and FS at later time points after sepsis onset in animal models, but whether Fer-1 could improve cardiac muscle strain in the late stage of CLP induced sepsis remains unknown.

In acute stage of sepsis, the hyper-activated immune response including the increased IL-6, IL-1β and TNFα, as well as immune cell infiltration are the major causes for the multiple organ damages (Zamora et al., 2018; Srdic et al., 2024). Activated immune cells mobilize into blood stream and infiltrate into multiple organs via chemokine axis (Chen et al., 2018). Increased neutrophil and macrophages infiltrated in heart tissue has been shown 6 h after LPS induced endotoxemia (Raeburn et al., 2001; Zhang et al., 2020). In our acute CLP induced sepsis model, increased neutrophil infiltration was found 6 h after CLP in myocardium but few macrophages were visualized as stained by CD68. This difference in infiltrated immune cell type could be explained by the different signaling between CLP and LPS model (Ullah et al., 2023), as LPS majorly acts via Toll-like receptor (TLR) pathways, while CLP-induced sepsis activates inflammation via STAT3 and cytokine expression (Ullah et al., 2023). Interestingly, Fer-1 pre-treatment significantly impaired myocardial inflammatory cytokine and chemokine expression, and dampened myocardial neutrophil infiltration at 6 h after CLP, suggesting that Fer-1 might systemically regulate neutrophil mobilization, recruitment and infiltration during CLP induced sepsis. The underlying mechanism might associate with decreased AP-1 (Jun/Fos) and NFκb (Singha et al., 2015) as suggested by the GO-TRRUST enrichment of transcriptional regulators in the Fer-1 treated CLP hearts. The underlying mechanisms warrant further investigation.

In conclusion, our study found that at early stage of CLP induced sepsis, cardiac dysfunction can be detected by load-independent cardiac muscle strain analysis. Myocardial ferroptosis signaling might not be the major driver for myocardial damage in early stage of sepsis. Fer-1 significantly protects the CLP mice partially via inhibiting myocardial neutrophil cell infiltration in early sepsis by directly impairing chemokine axis. This newly identified effect of Fer-1 treatment highlights its therapeutic potential in acute sepsis-induced cardiomyopathy.

## Methods

### Animals

All animal experiments were conducted in accordance with protocols (SYSU-IACUC-2024–002,534) approved by the Sun Yat-sen University Laboratory Animal Ethics Committee following the guidelines in the Guidelines for the Care and Use of Laboratory Animals. Ten to 12 weeks old male C57BL/6 mice were purchased from Guangzhou Ruige Biological Technology Co., LTD. Prior to the experiment, the mice were housed in SPF facility, given 12 h cycles of light and darkness, a temperature of 22 °C with a humidity of 60%, and unrestricted access to food and water for at least 1 week. Mice were randomly assigned to the Sham, CLP and Fer-1 group.

### CLP model and *in vivo* drug treatment

Under sterile conditions, mice were anesthetized by inhalation of 2% isoflurane. The cecum was ligated at 1 cm from the end of the cecum and punctured a single hole through the distal cecum with a 16-gauge needle to induce sepsis. The resuscitation of hyperkinetic stage of sepsis was induced by subcutaneous injection of 1 mL preheated 0.9% normal saline. Animals receiving sham operations had identical open procedures but without cecal ligation and puncture. Additionally, the mice in the CLP-Fer-1 group were treated with Fer-1 (Selleck Chemicals, S7243, 5 mg/kg) 2 h before surgery by i.p. injection, and corresponding CLP group were i.p. injected with vehicle for Fer-1.

### Echocardiography

Transthoracic echocardiography was performed on anesthetized mice 6 h after CLP surgery using the Vevo 3100 imaging system (Fujifilm) with a 30 MHz transducer. Heart rate and left ventricular (LV) dimensions, including diastolic and systolic wall thickness and left ventricular end-diastolic and end-systolic cavity dimensions, were measured from a two-dimensional long axis in the B and M modes at the papillary muscle level. he Doppler sampling volume cursor was placed above the mitral valve cusp to obtain the peak velocity of mitral valve flow E in early diastolic phase and peak velocity of mitral valve flow A in late diastolic phase using the four-cavity section from the apex of the heart. Videos from the long-axis imaging plane were used for cardiac muscle strain analysis. Cardiac functional parameters and strain analyses, including ejection fraction percentage (EF), fraction shortening (FS), left ventricular volume, and overall longitudinal strain (GLS), were calculated using the above primary measurements and accompanying software (Xiang et al., 2016; Xiang et al., 2017).

### Histological analysis

Cardiac tissues were fixed in 4% paraformaldehyde and embedded in paraffin. For immunohistochemistry (IHC), 5-μm paraffin sections were deparaffinized with xylene and rehydrated with the concentration gradient of ethanol. Then antigen retrieval was performed using a sodium citrate buffer (pH = 6.8) with a pressure-cooker. After that, sections were blocked with phosphate-buffered saline (PBS) containing 0.1% Triton-X100, 0.05% Tween20, 1% BSA and 5% goat serum. The following antibodies were used for immunostaining: Cd45 (1:100, CST, 70257S), Cd68 (1:200, CST, 97778S), Ly6G (1:100, Servicebio, GB11229), HRP conjugated Goat Anti-Rabbit IgG (H + L) (1:500, Servicebio, GB23303), Anti-rabbit IgG (H + L) (1:500, CST, 8889). For immunohistochemical staining, sections were incubated overnight at RT with primary antibodies followed by 3% H_2_O_2_ to inactivate endogenous peroxidase. Then, the sections were incubated with an HRP conjugated Goat Anti-Rabbit IgG reagent at 37 °C for 45 min followed by 3, 3′-diaminobenzidine (DAB, Servicebio, G1212) for color development, hematoxylin for nucleus display. Photos were obtained using an Eclipse E400 microscope (Nikon). For immunofluorescence staining, sections were incubated overnight at room temperature with primary antibodies followed by incubation with secondary antibodies for 1 h at RT. Nuclei were stained with Hoechst (1:10,000) for 10 min at RT. Fluorescent images were captured with Olympus Intelligent Microscope Model BX63 (61, 62).

### TUNEL staining

Paraffin sections from hearts 6 h post-sham or CLP surgeries were stained using a TUNEL kit (Beyotime, C1086) in accordance with the manufacturer’s instructions. The nuclei were visualized by Hoechst staining. Afterward, the samples were observed using Olympus Intelligent Microscope Model BX63: TUNEL-positive cells were green, and nuclei were stained as blue (Xiang et al., 2013).

### RNA isolation and real-time quantitative RT-PCR

For RNA isolation, heart tissues were collected and stored at −80°C. Sample RNA was isolated as previously described (Xiang et al., 2016; Xiang et al., 2017). Briefly, tissue samples were homogenized in 1 mL of TRIZOL reagent per 50–100 mg of tissue using 1 mm^3^ beads (EASYBIO, Cat# BE6860). 0.2 mL of chloroform per 1 mL of TRIZOL Reagent were added to extract RNA. RNA from each sample was precipitated from the aqueous phase by mixing with isopropyl alcohol. After washed with 75% ethanol, RNA pellet was dissolved in DEPC-treated water and the RNA concentration was measured. cDNA was then made from 1 µg RNA using PrimeScript™ RT reagent Kit with gDNA Eraser (TaKaRa), following manufacture’s instruction. Real time PCR assay was set up using TB Green^®^ Premix Ex Taq™ II (TaKaRa). The reaction was run in the Applied Biosystems™ QuantStudio™ using 1 µL of the cDNA and 0.4 µM primers in a 10 µL reaction according to the manufacturer’s instructions. The oligonucleotide primer sequences were as follows: 18s-F GCCGCTAGAGGTGAAATTCTT/18s-R CGT​CTT​CGA​ACC​TCC​GAC​T; mGpx4-F CCTCTGCTGCAAGAGCCTCCC/mGpx4-R CTT​ATC​CAG​GCA​GAC​CAT​GTG​C; mPtgs2-F GCGACATACTCAAGCAGGAGCA/mPtgs2-R AGT​GGT​AAC​CGC​TCA​GGT​GTT​G; mHmox1-F CACTCTGGAGATGACACCTGAG/mHmox1-R GTG​TTC​CTC​TGT​CAG​CAT​CAC​C; mSlc7a11-F CTTTGTTGCCCTCTCCTGCTTC/mSlc7a11-R CAG​AGG​AGT​GTG​CTT​GTG​GAC​A; mFth1-F GCCGAGAAACTGATGAAGCTGC/mFth1-R GCA​CAC​TCC​ATT​GCA​TTC​AGC​C; mHamp-F CAGCACCACCTATCTCCATCAAC/mHamp-R CAG​ATG​GGG​AAG​TTG​GTG​TCT​C; m-Il6-F TACCACTTCACAAGTCGGAGGC/m-Il6-R CTG​CAA​GTG​CAT​CAT​CGT​TGT​TC; m-Il1b-F TGGACCTTCCAGGATGAGGACA/m-Il1b-R GTT​CAT​CTC​GGA​GCC​TGT​AGT​G; m-Tnfa-F GATCGGTCCCCAAAGGGATG/m-Tnfa-R TGA​GGG​TCT​GGG​CCA​TAG​AA; m-Ccrl2-F GCCTCCATCTTCACGACAGTGT/m-Ccrl2-R GTG​AGG​TTT​GCC​AAA​GGC​ACA​C; m-CXCL2-F CATCCAGAGCTTGAGTGTGACG/m-CXCL2-R GGC​TTC​AGG​GTC​AAG​GCA​AAC​T; m-CXCL3-F TGAGACCATCCAGAGCTTGACG/m-CXCL3-R CCT​TGG​GGG​TTG​AGG​CAA​ACT​T; m-Cxcl5 -F CCGCTGGCATTTCTGTTGCTGT/m-Cxcl5 -R CAG​GGA​TCA​CCT​CCA​AAT​TAG​CG; m-Adamts1-F GAAGGCAAACGAGTCCGCTACA/m-Adamts1-R TTG​GGT​GTC​CAC​TCT​ACA​GTG​G; m-Adamts4-F GAACGGTGGCAAGTATTGTGAGG/m-Adamts4-R TTC​GGT​GGT​TGT​AGG​CAG​CAC​A; m-Adamts9-F TACCGAGAACCCAGTGGCGATT/m-Adamts9-R ACA​GGC​ACT​CTC​ATC​AGC​CAC​A; m-Mmp8-F GATGCTACTACCACACTCCGTG/m-Mmp8-R TAA​GCA​GCC​TGA​AGA​CCG​TTG​G; m-Cd93-F GATGGCTCTTTCTACTGCTCCTG/m-Cd93-R CCA​CAC​CTG​AAG​GAA​CCA​TCT​G; m-Cldn5-F TGACTGCCTTCCTGGACCACAA/m-Cldn5-R CAT​ACA​CCT​TGC​ACT​GCA​TGT​GC; m-Thbs1-F GGTAGCTGGAAATGTGGTGCGT/m-Thbs1-R GCA​CCG​ATG​TTC​TCC​GTT​GTG​A; m-Thbd-F GGAGAATGGTGGCTGTGAGTAC/m-Thbd-R GCA​CGA​TTG​AAC​CAC​AGG​TCT​TG. To quantify the mRNA expression levels, 18S was used as the internal reference and relative expression levels were calculated using the 2^−ΔΔCt^ method (Xiang et al., 2016; Xiang et al., 2017).

### Protein isolation and western blotting

The hearts were collected after sham or CLP. Tissues were homogenized in RIPA buffer (Beyotime, P0013E) with Protease inhibitor PMSF (Beyotime, ST506) and centrifuged at 12,000 rpm at 4° C for 20 min. The supernatants were collected. The protein concentration was determined using a BCA protein assay kit (Beyotime, P0010). 40 μg of protein was separated by 12.5% SDS-PAGE gel and transferred to PVDF membranes. After blocking in Intercept^®^ (TBS) Blocking Buffer (LI-COR, 927–60001), the blots were probed with primary antibodies: GPX4 (1:1000; ab12506), GAPDH (1:1000; 60004-1-Ig). After incubation with corresponding anti-mouse/rabbit secondary antibodies (1:10,000; Proteintech), immunoblots were visualized by using FDbio-Dura ECL Kit (Fdbio science, FD8020) and Amersham Image Quant800 (Cytiva). Signal intensities were quantified with ImageJ software. GAPDH was used as a loading control.

### Lipid peroxide assay

The lipid peroxide assay was performed according to the instructions of malondialdehyde (MDA) assay kit (Beyotime, S0131). The tissue homogenate was isolated as mentioned in protein isolation. Tissue homogenate samples were mixed with thiobarbituric acid and antioxidant reagent provided by the kit. After incubating the reaction system at 100° C for 15 min, the solution turned pink with accumulation of precipitate. All samples were then cooled down in water, and centrifuged at 1000g for 10 min. The supernatants were collected. The samples’ absorbance at 532 nm was measured by a plate reader (Varioskan LUX, Thermo Scientific). The concentration of MDA in tissue homogenate was quantified by the standard curve. The accurate calculation of MDA was based on the total amount of protein in each sample, which was tested using enhanced BCA protein assay kit (Beyotime, P0010) following the instructions of the manufacturer.

### Serum preparation, tissue lysate preparation and iron assay

The serum was collected from mice after CLP. The whole blood of mice was collected in tubes and was left undisturbed at room temperature for 30 min. Then samples were centrifuged at 12,000 rpm at 4°C for 10 min to remove clots. The serum was transferred into new tubes and stored at −80°C. The hearts were collected after CLP. Tissues were homogenized in Iron Assay buffer (Abcam, ab83366) with Protease inhibitor PMSF (Beyotime, ST506) and centrifuged at 12,000 rpm at 4°C for 20 min. The supernatants were collected. The protein concentration was determined using a BCA protein assay kit (Beyotime, P0010). The concentration of ferrous (Fe2^+^) and ferric (Fe3^+^) iron in serum and heart tissue were measured by Iron Assay kit (Abcam, ab83366). The iron assay was performed according to the instructions of the kit. Briefly, the serum or tissue lysate was mixed with iron probe. Then the mixture was incubated at 37 °C for 60 min protected from light. The samples’ absorbance at 593 nm was measured (Varioskan LUX, Thermo Scientific). The concentration of ferrous (Fe2^+^) iron and the concentration of total (Fe2^+^+Fe3^+^) iron were quantified by standard curve. For tissue, the accurate calculation of iron was based on the total amount of protein in each sample.

### RNASeq analysis

Six hours after surgery at the age of 8–12 weeks, total RNA from sham, CLP, Fer-1 group hearts (n = 2∼3 hearts/group) was isolated, and transcriptome profiles of hearts were analyzed using RNA-seq technology. Genes with low expression (FPKM ≤ 1 in both conditions and Read Counts ≤ 10 in both conditions) were excluded. ‘p-value < 0.05’ and ‘absolute log2 (fold change) > 1’ were used as the thresholds to judge the significance of gene expression differences. Differentially expressed genes were selected at the statistical significancy level. The visualization of DEGs was conducted via R (version4.3.2). Heatmap of Top 36 genes (based on FPKM ≥ 30) was conducted via R package ‘pheatmap’. GO Enrichment Analysis was conducted via R package ‘clusterProfiler’ (Wu et al., 2021) on the identified DEGs. Transcription factor to target analyses were performed using Metascape.com (version3.5.20240901) (Zhou et al., 2019).

### Statistical analysis

Data were expressed as mean ± SEM. All data sets were analyzed using GraphPad Prism (version 9.0). Statistical analyses between two groups were performed with Mann-Whitney U tests. For data with multiple groups, one-way ANOVA followed by Bonferroni’s multiple comparisons tests were performed. Kaplan Meier survival curves were analyzed by the Log-rank (Mantel-Cox) test. A 2-tailed p-value <0.05 was considered significant.

## Data Availability

The datasets presented in this study can be found in online repositories. The names of the repository/repositories and accession number(s) can be found in the article/Supplementary Material.
