# Supplementary figures and images for: Ferrostatin-1 improves acute sepsis-induced cardiomyopathy via inhibiting neutrophil infiltration through impaired chemokine axis
Source: Front Cell Dev Biol. 2024 Dec 12;12:1510232. doi: 10.3389/fcell.2024.1510232 (PMC11669711; doi:10.3389/fcell.2024.1510232)

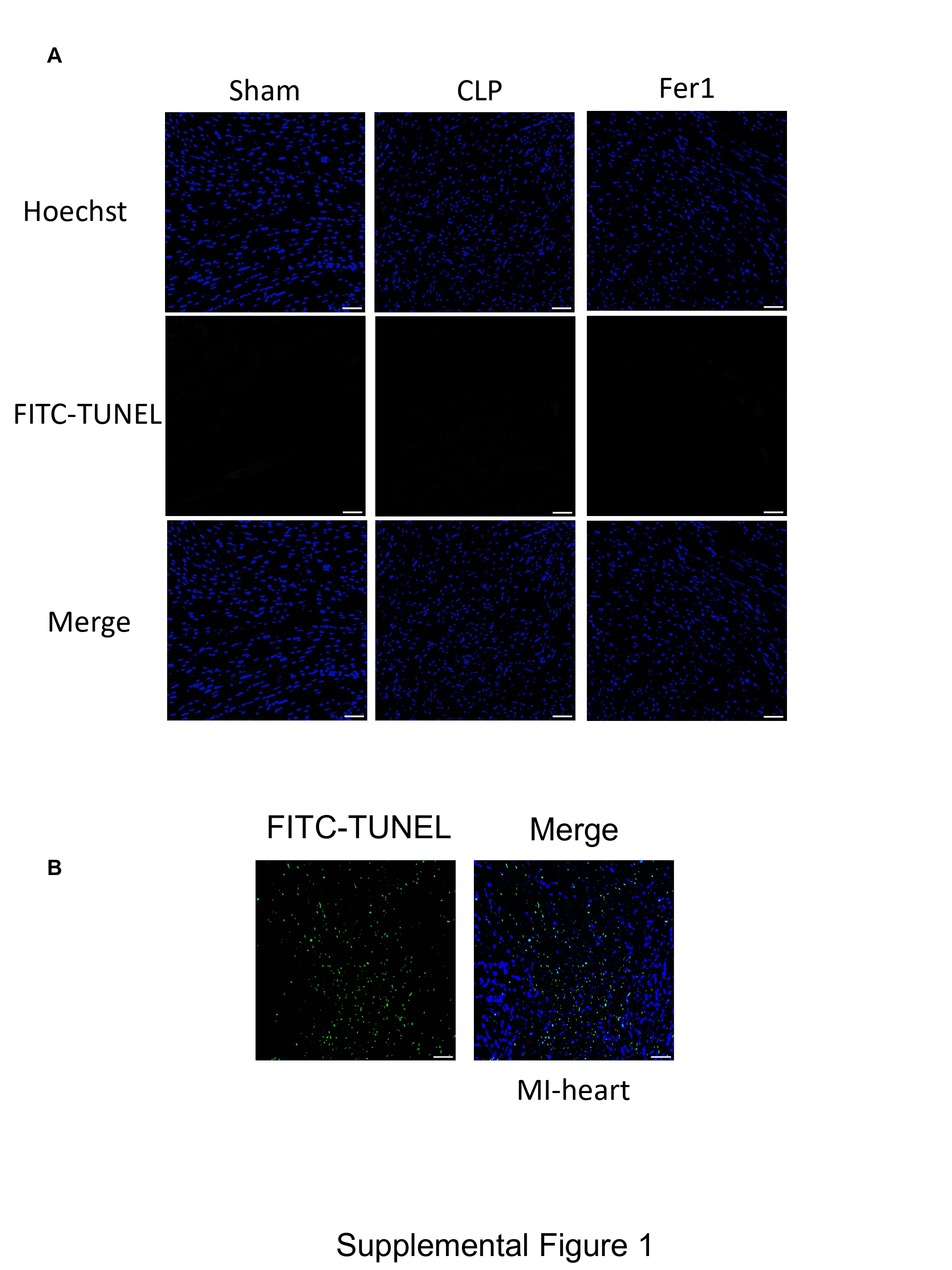

Supplement: Supplementary file 4 [file Image1.jpeg]

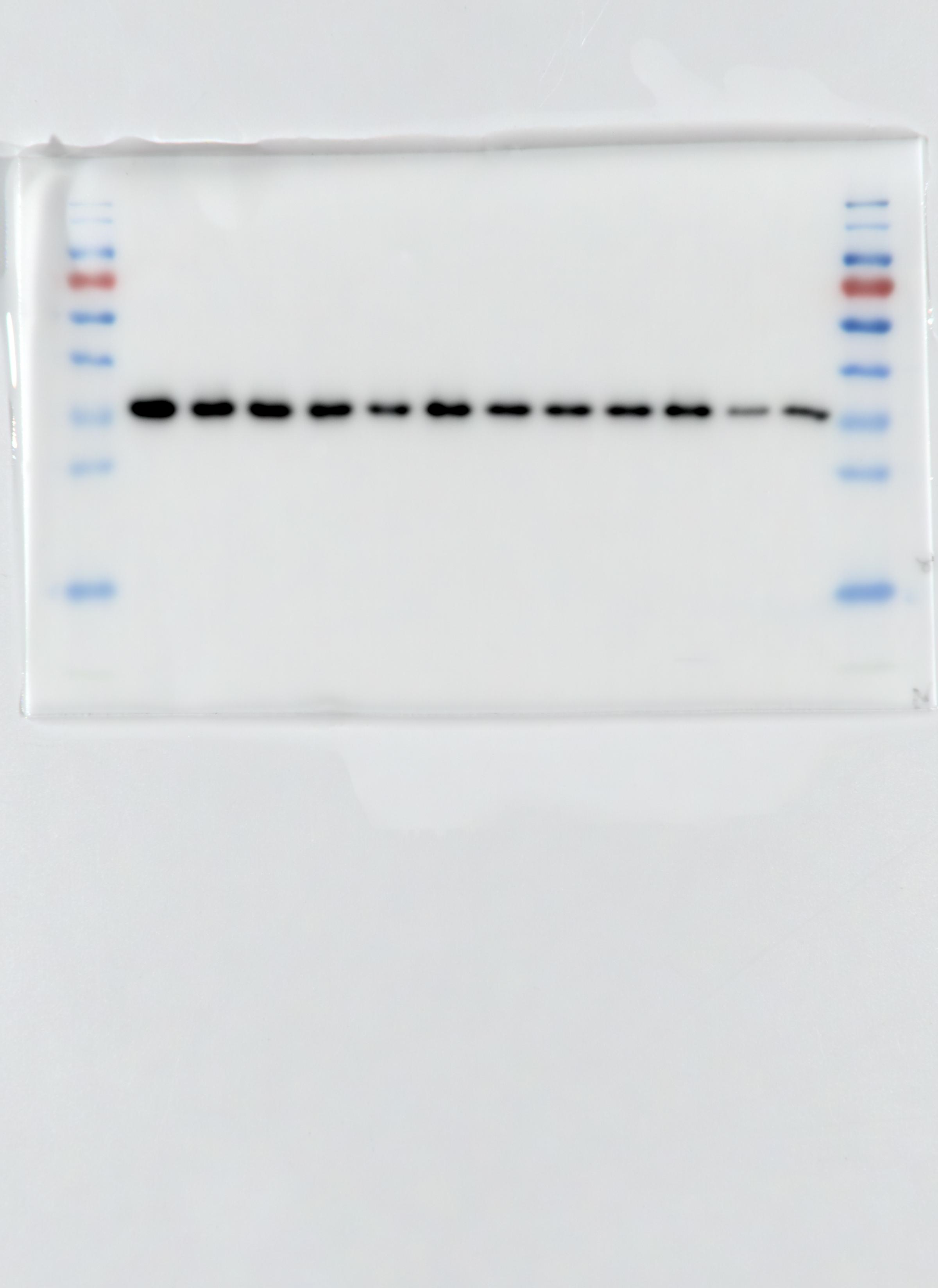

Supplement: Supplementary file 5 [file DataSheet1.zip › WB-tif/heart-gapdh 20240710_122304_Ch_Chemi+Marker.tif]

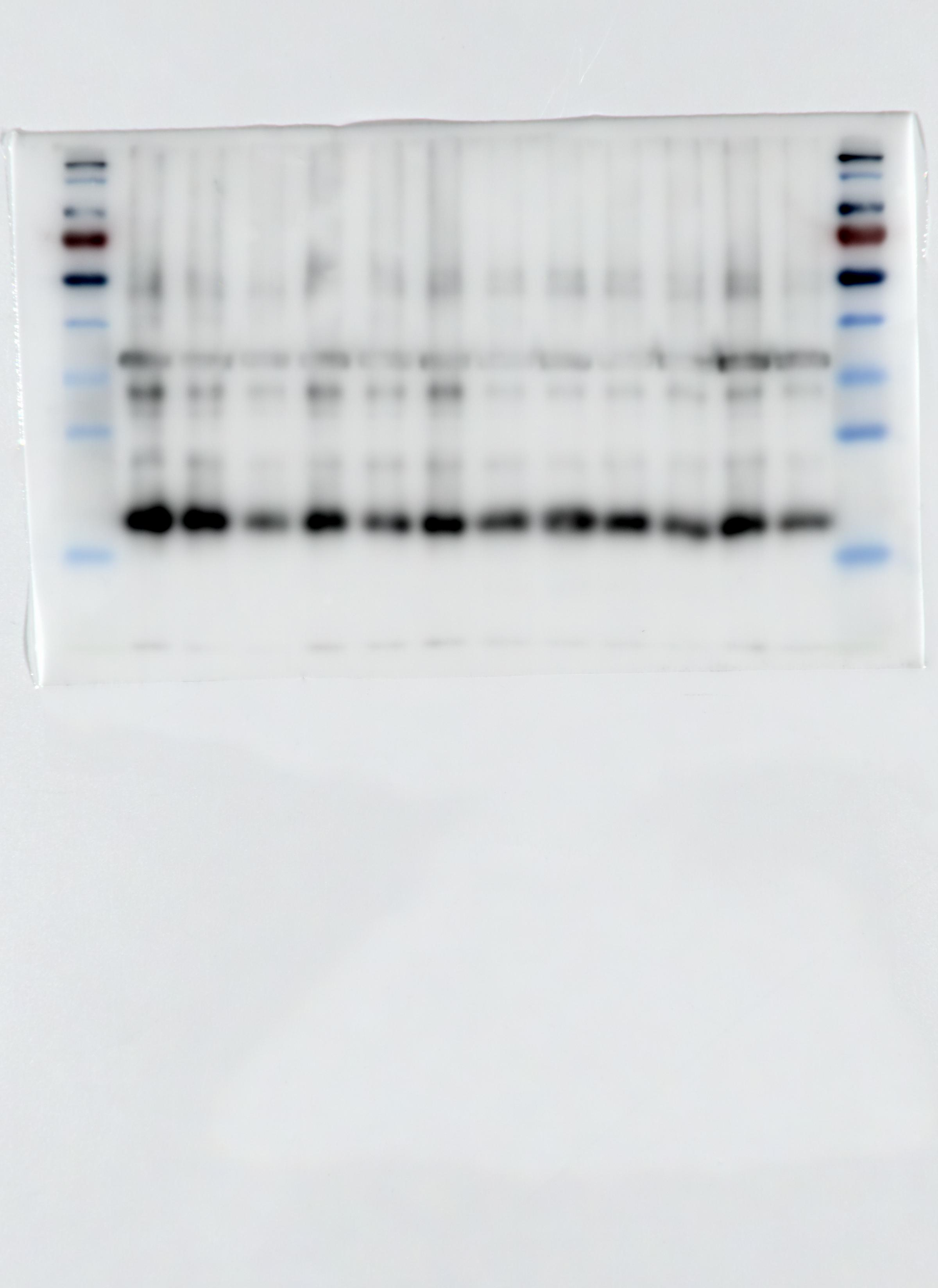

Supplement: Supplementary file 5 [file DataSheet1.zip › WB-tif/heart-gpx4 20240710_123805_Ch_Chemi+Marker.tif]

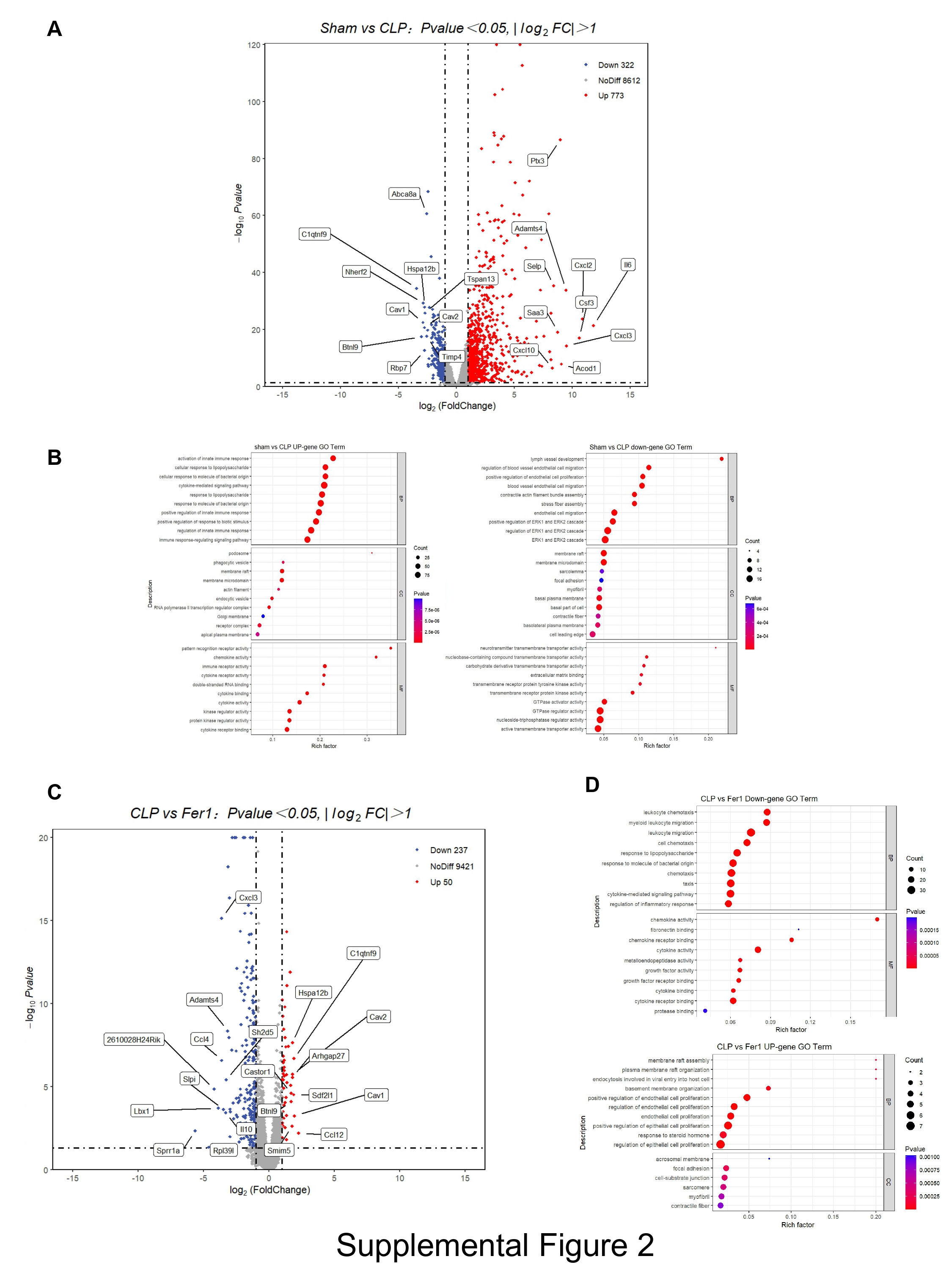

Supplement: Supplementary file 6 [file Image2.jpeg]
